# Virulence and resistance features of *Pseudomonas aeruginosa* strains isolated from chronic leg ulcers

**DOI:** 10.1186/s12879-016-1396-3

**Published:** 2016-03-08

**Authors:** Mihaela Georgescu, Irina Gheorghe, Carmen Curutiu, Veronica Lazar, Coralia Bleotu, Mariana-Carmen Chifiriuc

**Affiliations:** Dermatology Department, Central University Emergency Military Hospital Dr Carol Davila, Bucharest, Romania; Microbiology Department, Faculty of Biology, University of Bucharest, Bucharest, Romania; Research Institute of the University of Bucharest -ICUB, Bucharest, Romania; Ștefan S Nicolau Institute of Virology, Romanian Academy, 030304 Bucharest, Romania

**Keywords:** *Pseudomonas aeruginosa*, Genetic and phenotypic virulence factors, Chronic leg ulcers

## Abstract

**Background:**

The purpose of this study was to evaluate the virulence profiles of *Pseudomonas aeruginosa* clinical strains recently isolated from patients hospitalized for chronic leg ulcers in the Dermatology Department of Central Military Emergency University Hospital “Carol Davila”, Bucharest, Romania.

**Methods:**

The phenotypic screening evaluated eight soluble virulence factors (haemolysins, lecithinase, lipase, caseinase, gelatinase, amylase, DNase, aesculin hydrolysis), as well as adherence ability (Cravioto adapted method) and invasion capacity on HeLa cells (gentamicin protection assay). Seven virulence genes encoding for protease IV, 3 exoenzymes (exoS, exoT, exoU), two phospholipases plcH- haemolytic phospholipase C and plcN- non-haemolytic phospholipase C) and alginate were investigated by PCR.

**Results:**

The pore forming toxins and enzymes were expressed in variable proportions, the majority of the tested strains producing beta haemolysin (92.3 %), lipase (76.9 %) and lecithinase (61.5 %). The most frequent virulence genes detected in the analyzed strains were the *ExoT* (100 %) and *AlgD* (92.3 %) genes, genes codifying for phospholipases (84.6 % each of them) and for protease IV (61.5 %).

**Conclusions:**

This study reveals that correlating virulence profiles and infection clinical outcome is very useful for setting up efficient preventive and therapeutic procedures for hospitalized patients with chronic leg ulcers and positive *P. aeruginosa* cultures.

## Background

Chronic leg ulcers represent a clinically highly relevant topic, this condition affecting 1–2 % of the general population and being responsible for increased morbidity and impaired quality of life. *Pseudomonas aeruginosa*, one of the most common bacteria isolated from chronic wounds [1], is an opportunistic pathogen with innate resistance to many antibiotic classes, including antipseudomonal penicillins, carbapenems, aminoglycosides and ciprofloxacin [2, 3]. Treatment options for infections caused by antibiotic resistant *P. aeruginosa* is limited to carbapenems, but increasing resistance rates mediated in most cases by the production of carbapenemases such as metallo-β-lactamases (MBLs) are reported [4, 5]. In case of *P. aeruginosa* isolated from chronic wounds there is no consensus on the first-line antibiotic to be used [6].

Besides intrinsic and acquired resistance to many antibiotics, *P. aeruginosa* expresses many extracellular virulence factors (exotoxins S and T with ADP-ribosylating activity which cause disruption of the host cell cytoskeleton, cytotoxin ExoU with phospholipase activity and cytotoxin Exo Y with an adenylate cyclase activity, phospholipases, protease IV and elastase) and surface associated components, as alginate. All these factors contribute to massive tissue damage, blood infection dissemination, and progression of the disease, and also limit treatment options with currently available antibiotics [7, 8]. A retrospective multicenter evaluation of 970 patients with chronic leg ulcer treated in different dermatologic wound care centers from Germany revealed that *P. aeruginosa* was found in 31.1 % of the analyzed cases and a positive correlation regarding wound size and duration was observed [9]. Because in many cases chronic leg ulcers are associated with diabetes, and taking into account the high resistance of this species, persistence of *P. aeruginosa* in these patients was observed. In a longitudinal survey regarding patients with non-healing venous ulcers, *P. aeruginosa* was isolated ≥ 3 years from the same patients, and a longer wound duration compared to all analyzed patients was revealed [10].

Considering the above mentioned aspects and taking into account the lack of such studies on Romanian strains the purpose of this study was to investigate the phenotypic and genotypic profile of *P. aeruginosa* strains isolated from Romanian patients with chronic leg ulcers.

## Methods

### Isolation and identification of bacterial strains

This study was conducted on a total of 12 *P. aeruginosa* clinical strains isolated during July 2013 - June 2015, from 130 patients hospitalized for chronic leg in the Department of Dermatology of the Central Military Emergency University Hospital “Carol Davila”, Bucharest, Romania. The patient or the legally authorized representative had to be able to read and sign the informed consent. Swab samples were collected for aerobic bacterial culture. The swabs were transported in thioglycolate broth media and then transferred on 5 % sheep blood agar. Cultures were incubated at 37 °C aerobically for 24 h. The identification of microbial isolates was performed using Gram staining, conventional biochemical tests and API BioMerieux systems (20NE).

### Evaluation of the soluble enzymatic factors

The virulence phenotypes were assessed by performing enzymatic tests for the expression of some soluble virulence factors. Overnight culture of the strains was evaluated for the following virulence factors expression: haemolysins, other pore forming toxins (lecithinase, lipase), proteases (caseinase, gelatinase), amylase and aesculin hydrolysis. Detection of *haemolysin* production was performed by spotting the fresh cultures on 5 % sheep blood agar medium and incubation at 37 °*C* for 24 h. The colorless area around the culture revealed the presence of haemolysis activity. For *lipase* production the strains were spotted on 1 % Tween 80 agar as a substrate and followed by incubation at 37 °C for 24 h. An opaque (precipitation) zone around the spot was registered as positive reaction; for *lecithinase* production, the cultures were spotted into 2.5 % yolk agar and incubated at 37 °C for 24 h. A clear zone around the spot indicated the lecithinase production. The protease activity (*caseinase* and *gelatinase*) was determined using 15 % soluble casein agar, respectively 3 % gelatin as substrate. The strains were spotted and after incubation at 37 °C for 24 h, a white precipitate surrounding the growth indicated casein proteolysis, and colorless area around culture due to the gelatin hydrolysis, indicated the positive reaction for gelatinase. *Amylase* was detected using agar with 1 % starch and hydrolysis was revealed after adding Lugol's solution (yellow ring around the culture, while the rest of the plate will be blue). For the *aesculin hydrolysis* the medium containing Fe ^3+^ citrate was used and inoculated by spotting, then incubated for 24 h at 37 °C. A black precipitate around culture due to esculetol released under the action of beta-galactosidase was considered positive reaction.

**Evaluation of biofilm development on inert substrata** was assessed by the microtiter method. Overnight bacterial cultures were grown in 96 multi-well plates containing Tryptic Soy Broth (TSB) for 24 h at 37 °C, then the plate was emptied and washed three times with phosphate buffered saline (PBS). The adherent cells were then fixed with cold methanol, stained with an alkaline 1 % violet crystal solution for 15 min, washed with water and resuspended in a 33 % acetic acid solution. The intensity of the suspension was spectrophotometrically assessed, the amount of adhered biomass being proportional to the absorbance value read at 492 nm.

### Evaluation of the bacterial adherence to cellular substrata (HeLa cells)

For the adherence assay, Cravioto’s adapted method was used [11, 12]. Briefly, HeLa cell monolayers were washed with PBS and 1 ml of fresh medium without antibiotics was aseptically added to each well. The suspensions of *P. aeruginosa* obtained from mid-logarithmic phase cultures grown in nutrient broth were adjusted to 10^7^ colony forming units (CFU)/ml and 1 ml was used for the inoculation of each well. After two hours of incubation at 37 °C the monolayers were washed 3 times with PBS, fixed in ethanol (3 min) and stained with 1:10 v/v Giemsa solution for 20 min. The plates were washed, dried at room temperature overnight, and examined by optic microscopy using wet objective (×1000 magnification), in order to evaluate the adherence indexes and *patterns*. The adherence indexes were expressed as the ratio between the number of the eukaryotic cells with adhered bacteria and 100 eukaryotic cells counted on the microscopic field. The adherence *patterns* were defined as: localized adherence (LA) when tight clusters of microorganisms were noticed on the HeLa cell surface, aggregative adherence (AA) when a microbial stacked brick pattern characterized the attachment, diffuse adherence (DA) when the bacteria adhered diffusely, covering the whole surface of the cell [13].

### Evaluation of the bacterial invasion capacity

To determine the invasion ability the antibiotic protection assay was used [14, 15]. Bacterial suspensions were inoculated in duplicate on HeLa cells grown in 6-well plates and incubated at 37 °C for two hours. The infection assay was performed in the same manner as the adherence assay, but after the incubation period in one of two wells inoculated with each strain 100 μg/ml gentamicin was added in order to kill extracellular bacteria. The plates were further incubated for another hour; after incubation, plates were washed 3 times with PBS and the HeLa cells were permeabilized with 0.1 % Triton X-100 for 10 min, at 37 °C. Serial dilutions of suspended cells harvested were seeded on nutritive agar (three technical replicates for each dilution) in order to establish the invasion indexes, calculated as CFU/ml.

### Evaluation of the antibiotic susceptibility

The antibiotic susceptibility testing was performed by Kirby-Bauer standard disk diffusion method (panels of antibiotic disks recommended by CLSI, 2013, 2014, 2015).

### PCR assays for virulence genes detection

The genetic support of the virulence factors was investigated by simplex and multiplex PCR, using a reaction mix of 20 or 25 μl (PCR Master Mix 2x, Thermo Scientific) containing 1 μl of bacterial DNA extracted using the alkaline extraction method. In this purpose, 1–5 colonies of bacterial cultures were suspended in 1.5 ml tubes containing 20 μl solution of NaOH (sodium hydroxide) and SDS (sodium dodecyl sulphate) and heated on a thermoblock at 95 °C for 15 min. for the permeabilization of bacterial wall. The following step was the addition of 180 μl of TE buffer (TRIS + EDTA) 1X and centrifugation at 13000 rpm for 3 min. All PCR reactions were performed using the Thermal Cycler machine Corbet. Genomic DNA was used as a template for the PCR screening of 7 virulence genes encoding for protease IV, three exoenzymes – exoS, exoT, exoU, two phospholipases - plcH (haemolytic phospholipase C) and plcN (non-haemolytic phospholipase C) and for alginate. The PCR reactions were initiated with 1 cycle at 95 °C for 2 min, followed by 30 cycles at 94 °C for 30 s, 58 °C for 30 s, 72 °C for 1 min and a final elongation step at 72 °C for 7 min. The amplification products were visualized by electrophoresis on a 1 % agarose gel, stained with the specific weight marker (100pb, Ladder Bench Top, Promega, USA).

## Results and discussion

In the present study, from 130 bacterial strains isolated from chronic leg ulcers of hospitalized patients, only 12 (9.23 %) were *P. aeruginosa,* accounting for a smaller percentage comparing with the number of cases reported in other studies. For example, in a study of Wolcott et al. (2015) performed on 2,963 patients with chronic wounds, *P. aeruginosa* represented 25 % from the total number of the isolated strains [16]*.*

**Fig. 1:**
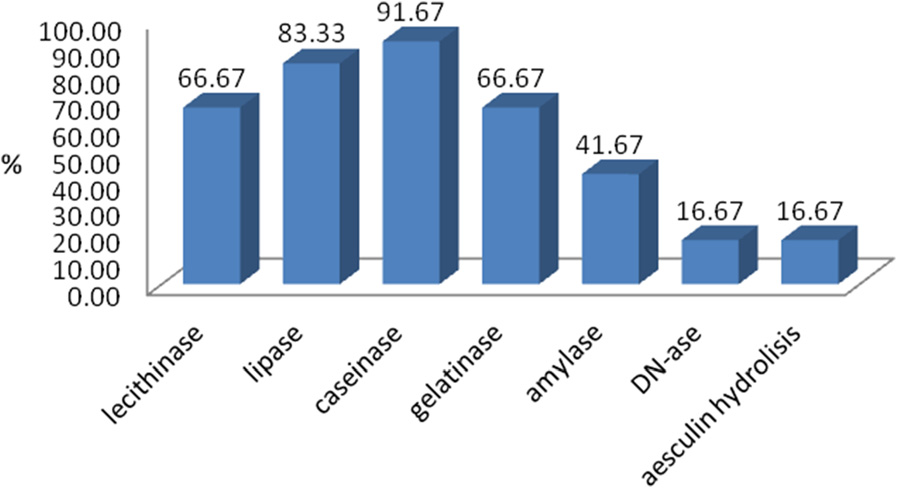
Graphic representation of enzymatic virulence factors expression

The isolated strains expressed between one and six of the seven investigated soluble virulence factors (Table 1). The most frequently expressed soluble virulence factors were caseinase (91.67 %) followed by beta haemolysins and lipase (83.33 %) (Fig. 1). Production of proteases by the majority of the tested strains indicates the ability of these strains to induce tissue lesions and to delay the wound healing. The pore forming enzymes, i.e. beta haemolysins, lecithinase (66.67 %) and lipase play an important role in dissemination of infection and wound extent. Although some studies [17] reported an increased expression of DNase in the strains isolated from wound secretions, in our study only 2 of the 12 strains of *P. aeruginosa* produced DNase (16.67 %).

**Table 1:** Phenotypic and molecular characteristics of *Pseudomonas aeruginosa*strains

Strain no.	Strain	DNase	Aesculin hydrolysis	Amylase	Caseinase	Lecithinase	Lipase	Gelatinase	Haemolysin	PLCH	PLCN	ExoU	ExoT	AlgD	ExoS	TCF/TCR
1	1333(1)	-	**+**	**+**	**+++**	**+**	**+**	**+**	**β**	**+**	**+**	**-**	**+**	**+**	**+**	**+**
2	371	-	**+**	**+**	**+++**	**+**	**+**	**+**	**β**	**+**	**+**	**-**	**+**	**+**	**+**	**+**
3	c10	-	-	**+**	**+++**	**+**	**+**	**+**	**β**	**+**	**+**	**-**	**+**	**+**	**+**	**+**
4	1601(1)	**+**	-	**+**	**+++**	**+**	**+**	**+**	**β**	**+**	**+**	**-**	**+**	**+**	**+**	**+**
5	1453(1)	-	-	-	**+++**	+	**+**	**+**	**β**	**+**	**+**	**-**	**+**	**+**	**-**	
6	2469(1)	-	-	-	**+++**	**+**	**+**	**+**	**β**	**-**	**-**	**+**	**+**	**-**	**-**	**-**
7	5711(1)	**+**	-	**+**	**+++**	-	**+**	**+**	**β**	**+**	**+**	**-**	**+**	**+**	**+**	**+**
8	R2	-	-	-	**+++**	-	**+**	**+**	**β**	**+**	**+**	**-**	**+**	**+**	**-**	**+**
9	c03	-	-	-	**+++**	-	**+**	-	**β**	**+**	**+**	**-**	**+**	**+**	**-**	**-**
10	2576(1)	-	-	-	**+++**	-	**+**	-	**β**	**+**	**+**	**-**	**+**	**+**	**-**	**+**
11	DU	-	-	-	**+**	**+**	-	-	δ	+	+	-	+	+	+	-
12	DR	-	-	-	-	**+**	-	-	δ	-	-	-	+	+	-	+

**Fig. 2:**
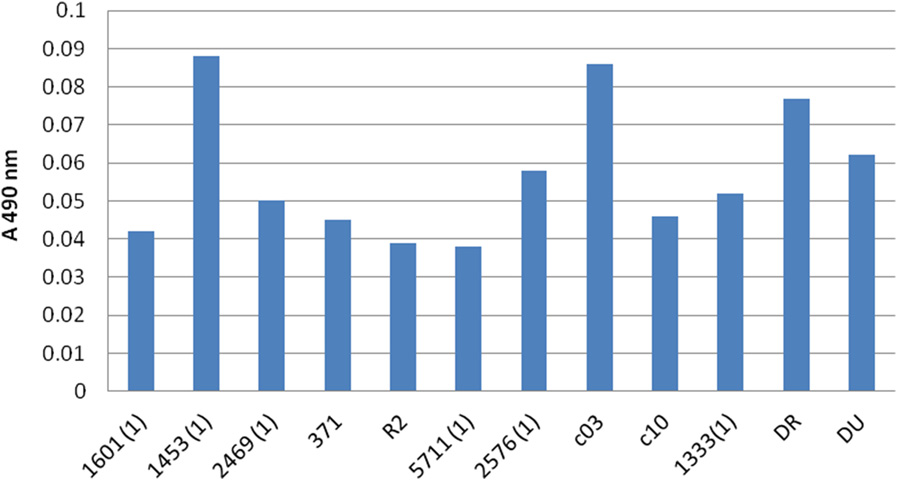
Graphic representation of adherence to inert substratum: all strains exhibited adherence capacity, the highest level being observed in the case of 1453(1), c03 and DR strains. The number of adherent bacteria was determined by measuring the absorbance at 490 nm

Regarding the cell-associated virulence factors, the analyzed strains showed variable rates of adherence to the inert substratum (Fig. 2). This adherence phenotype is correlated with the high capacity of *P. aeruginosa* strains to develop complex biofilm structures [18], aspect that can explain the persistence of this bacterium in the organism, and also its high antibiotic resistance.

**Fig. 3:**
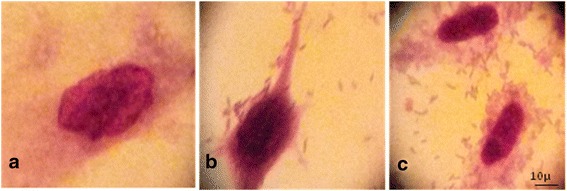
Different adherence patterns of *P. aeruginosa* analyzed strains: **a**- control, **b**- localized pattern, **c**- aggregative pattern (1000X)

*P. aeruginosa* analyzed strains exhibited different indexes of adherence to the cellular substratum represented by HeLa cells, ranging from 12 to 100 %. All the adherence patterns to HeLa were observed, but mostly the localized *pattern* was revealed (Fig. 3).

**Fig. 4:**
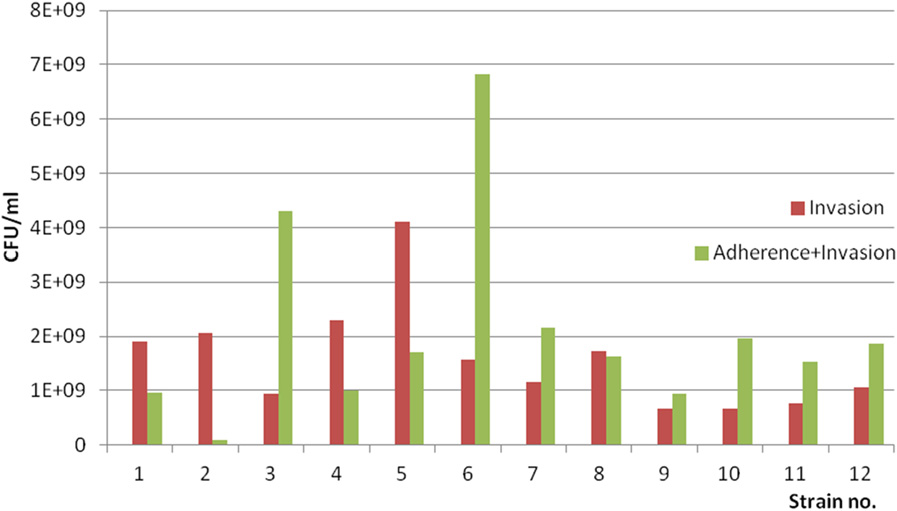
Graphic representation of adherence and invasiveness rates of *P. aeruginosa –*the number of colony forming units of adherent and invasive bacteria (green) and invasive bacteria (treated with gentamicin) (red) - all the strains adhered to the cellular substrate and also succeeded to invade the cells, some of them showing a high invasive capacity (strains no 1, 2, 4 and 5)

The analyzed strains proved the ability to invade the cellular substratum, which was somehow expected due to the presence of pore forming enzymes that cause pores in the cell membranes, and facilitate internalization of the bacteria in the host cells (Fig. 4, Table 1).

The ability of *P. aeruginosa* to survive in host cells may explain some aspects of host-pathogen relationship. The ability of bacteria to invade eukaryotic cells could protect them against host defense and antibiotic treatment facilitating the recurrent chronic diseases and long-term colonization process.

**Fig. 5:**
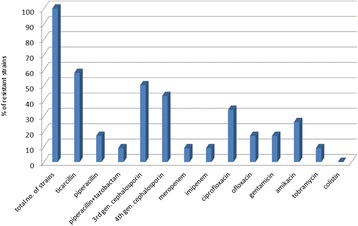
Antibiotic resistance profiles (%) among isolated *Pseudomonas* strains

*P. aeruginosa* strains isolated from chronic leg wound were resistant in high proportions to ticarcillin, to third and fourth generation cephalosporins and to ciprofloxacin. Only one strain demonstrated resistance to imipenem and meropenem (Fig. 5). None of the isolated strains demonstrated resistance to colistin. A recent study in Romania [18] identified 4 strains of *P. aeruginosa* showing comparable resistance rates to antibiotics.

On the other hand, international data demonstrate variable resistance: one study revealed strains isolated from chronic wounds resistant to piperacillin/tazobactam and aztreonam [18], another study found *P. aeruginosa* strains 100 % susceptible only to imipenem and meropenem, followed by ceftazidime (86 %) and amikacin, aztreonam, cefepime, ciprofloxacin, and piperacillin tazobactam (57 % each) [19]; opposite, another study found only 11.5 % resistance to imipenem (out of a collection of 512 *P. aeruginosa* strains), 66 % of the strains resistant to streptomycin, and 25.2 % and 28.5 % respectively were resistant to aminoglycoside antibiotics ciprofloxacin and gentamicin, respectively, polymyxin B having the greatest antibacterial activity (4.9 % resistant strains to this antibiotic) [20].

**Fig. 6:**
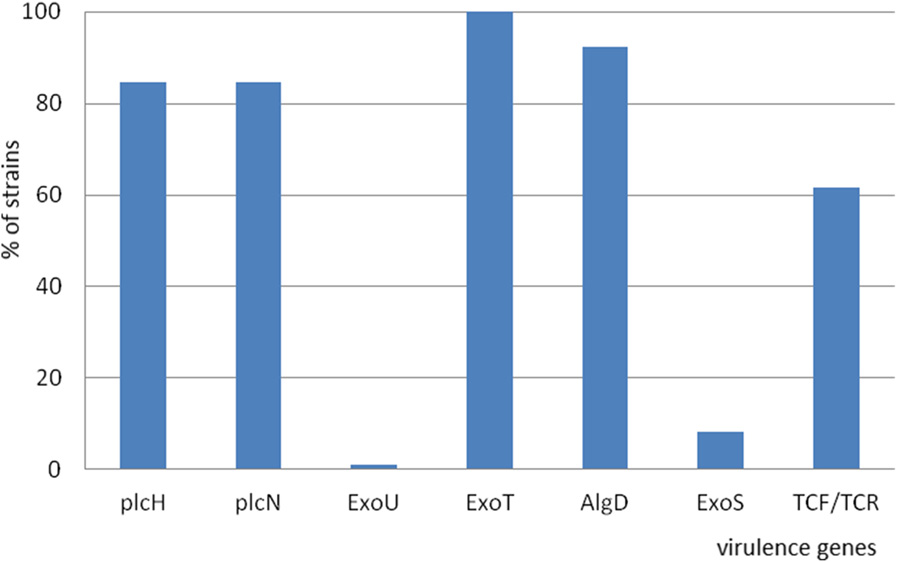
Percentage distribution of virulence genes in analyzed strains

The molecular analysis through PCR arrays showed that all the analyzed strains revealed the *ExoT* gene, 92.3 % of the isolates expressed *AlgD* gene, 84.6 % of the strains revealed *plcH* and *plcH* genes, 61.5 % of *P. aeruginosa* expressed the gene codifying for protease IV (*TCF/TCR)* and only 1 strain expressed the *ExoU* gene (Fig. 6).

**Fig. 7:**
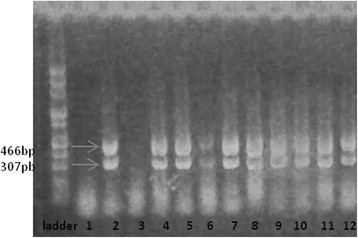
Electrophoresis gel for *PlcH (466pb)* an*d PlcN (307pb)* genes: all the tested strains except no. 2469 and DR revealed the two phospholipases. Lines: PCR Marker (Promega) - 100pb, 1 - 2469, 2- DU, 3- DR, 4- 571(1), 5- 1453, 6- C03, 7- 1333, 8- 2576, 9- 371, 10-C10, 11- 601, 12- R2

Multiple bacterial virulence factors impact the pathogenesis of *P. aeruginosa* infections. The combination of virulence factors expressed by each *P. aeruginosa* strain tends to determine the outcome of an infectious process. In the hospitals units, it is often difficult to distinguish between colonization and infection, and no diagnostic tool is available to assess the virulence potential of a given isolate [21, 22]. Among *P. aeruginosa* isolates three soluble proteins are responsible for invasion: a rhamnolipid and two phospholipases C (haemolytic phospholipase C (plcH), and non-haemolytic phospholipase C (plcN)) [23]. These two phospholipases could work synergistically, plcH would promote degradation of the erythrocyte membrane (phospholipids components of the outer leaflet: phosphatidylcholine and sphingomyelin), exposing the inner leaflet. Plc-N could then hydrolyze phosphatidylserine present in the inner leaflet. Our results of genotyping analysis showed that 84.6 % of the *P. aeruginosa* isolates possess phospholipases genes (Figs. 6 and 7) compared with other studies from our country in which only the plcH gene was revealed in the strains isolated from blood cultures and wound secretions [17]. The phospholipase gene expression is sustained at phenotypic level, the same strains being positive for lipase production.

**Fig. 8:**
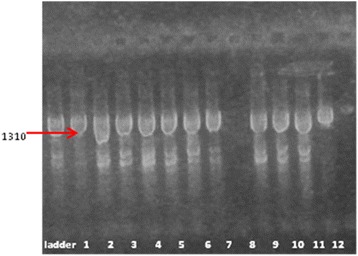
Electrophoresis gel of AlgD gene. The figure shows that all the analyzed strains except one (the strain DR) are positive for the respective gene. Lines: PCR Marker (Promega) - 100pb, 1-R2, 2-1333(1); 3-5711, 4-1453, 5-2576, 6-C10, 7-2469, 8-DR, 9-1601, 10-371, 11-C03, 12-DU

Our PCR results concerning the presence of *algD* gene showed that 92.3 % of the isolates express this gene (Fig. 8). The *algD* gene encodes GDP-mannose dehydrogenase, which acts as a rate-limiting enzyme in mucoid strains by catalyzing the conversion of GDP-mannose to GDP-mannuronic acid, thereby promoting the cell to alginate production [24], which demonstrates the involvement of these strains in infections with biofilm formation [23]. And indeed, the high level of *AlgD* gene expression correlates with phenotypic results, adherence to inert substratum being also observed among tested strains (Table 1).

**Fig. 9:**
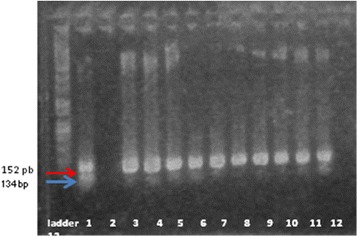
Electrophoresis gel of *ExoU and ExoT genes*: the figure shows that all the isolates revealed the ExoT. 2469 expressed the ExoU gene. Lines: PCR Marker (Promega) - 100pb, 1-2469, 2-DR, 3-5711, 4-1453, 5-C03, 6-1333(1), 7-2576, 8-371, 9-C10, 10-R2, 11-DU, 12-1601

**Fig. 10:**
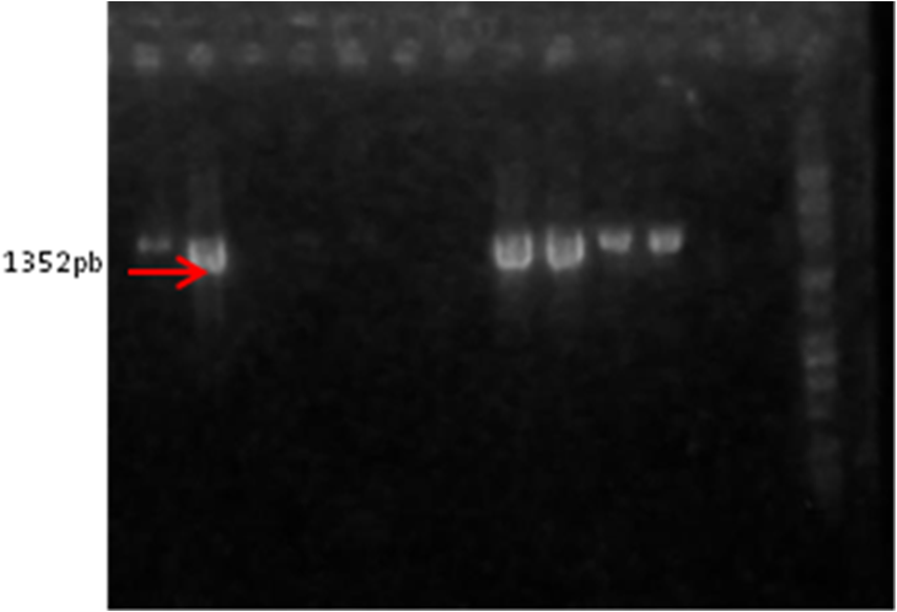
Electrophoresis gel of *ExoS gene:* the positive strains are 1333, 5711, 1601, 371, c10, DU. PCR Marker (Promega) - 100pb, 1-R2, 2-1333(1); 3-5711, 4-1453, 5-2576, 6-C10, 7-2469, 8-DR, 9-1601, 10-371, 11-C03, 12-DU

It is known that the exotoxins ExoS, ExoY, and ExoT inhibit invasion, while ExoU confers cytotoxicity [25–27]. ExoT and ExoS act on a number of host small G proteins, thus altering the cytoskeleton and signaling pathways [28]. The results of Shaver and Hauser indicate that ExoU has the greatest effect on virulence of the type III secreted proteins [29]. The concomitant presence of ExoU and ExoS was also revealed by one isolate of *P. aeruginosa* (Figs. 9 and 10).

**Fig. 11:**
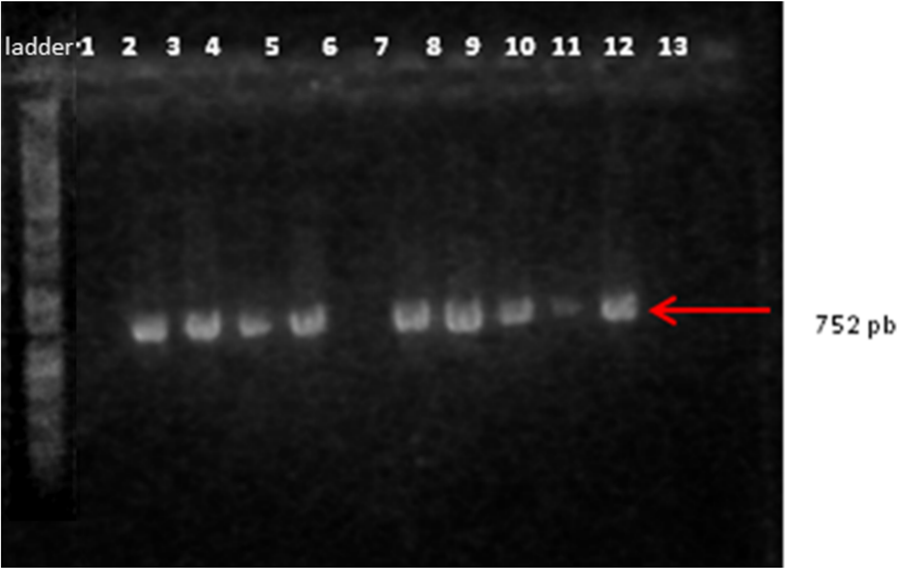
Electrophoresis gel of *protease IV (TCF/TCR)* the positive strains are no. 1333, 371, C10, 2576, 5711, R2, 1601, DR. Lines: PCR Marker (Promega) - 100pb, 1-1453, 2-DU, 3-2576, 4-5711, 5-R2, 6-1601, 7-2469, 8-DR, 9-1333(1), 10-371, 11-C03, 12-C10

The isolated strains of *P. aeruginosa* exhibited a high frequency of protease IV (TCF/TCR), which plays a role in tissue injuries (Fig. 11). Holban A.M. et al. [17] reported the highest frequency of plcH and protease IV genes in *P. aeruginosa* strains analyzed in their study.

## Conclusions

Our phenotypic and molecular results demonstrate that the analyzed *P. aeruginosa* strains express virulence features which explain the survival and implication of these strains in the poor prognosis of chronic leg wounds. The phenotypic virulence markers were correlated with some specific virulence genes profiles, revealing that the isolates could adapt easily to the microenvironment encountered within the host by modulating the expression of these genes. Furthermore, the diversity of virulence factors expression explains the large panel of clinical manifestations in *Pseudomonas aeruginosa* infections. All the data resulting from this study, the first of its kind in our country, could help clinicians to achieve correlations between clinical manifestations and the virulence of the involved strain and to adjust the therapeutic approach, consequently.
